# Living with orofacial conditions: psychological distress and quality of life in adults affected with Treacher Collins syndrome, cherubism, or oligodontia/ectodermal dysplasia—a comparative study

**DOI:** 10.1007/s11136-014-0826-1

**Published:** 2014-10-25

**Authors:** Amy Østertun Geirdal, Solfrid Sørgjerd Saltnes, Kari Storhaug, Pamela Åsten, Hilde Nordgarden, Janicke Liaaen Jensen

**Affiliations:** 1 Faculty of Social Sciences, Oslo and Akershus University College of Applied Sciences, Oslo, Norway; 2 TAKO-Centre, Lovisenberg Diakonale Hospital, Oslo, Norway; 3 Department of Oral Surgery and Oral Medicine, Faculty of Dentistry, University of Oslo, Oslo, Norway

**Keywords:** Psychological distress, Quality of life, Treacher Collins syndrome, Cherubism, Oligodontia/ectodermal dysplasia

## Abstract

**Purpose:**

The relationship between quality of life, psychological distress, and orofacial syndromes in children and adolescents has been reported in several studies. However, little is known about differences in psychological distress and quality of life among adults with different orofacial conditions. Therefore, the aims of this study were to examine and compare these factors among three groups of adults affected by Treacher Collins syndrome (TCS), cherubism, and oligodontia/ectodermal dysplasia (ED).

**Methods:**

We included 11 individuals with TCS (mean age 46.9, SD 12.9 years), 15 with cherubism (mean age 50.3, SD 16.8 years), and 49 with oligodontia/ED (mean age 30.7, SD 15.6 years). The respondents completed questionnaires related to psychological distress and quality of life.

**Results:**

The oligodontia/ED group had a significantly higher level of anxiety and worse mental health-related quality of life than both the TCS and cherubism groups. Adults with TCS reported the highest level of depression, and the lowest levels of overall quality of life, well-being, and physical health-related quality of life. The cherubism group displayed the best overall quality of life, well-being, and mental health.

**Conclusions:**

Psychological distress and quality of life differed in various orofacial conditions. This study provided insight into these aspects that may contribute to improved care.

## Introduction

Treacher Collins syndrome (TCS), cherubism, oligodontia, and ectodermal dysplasias (EDs) are all examples of rare genetic conditions that affect the orofacial complex. These conditions may affect the head, face, mouth, or neck region, and they may influence both appearance and function. The types of tissues affected and the level of severity vary among different diagnoses, and also among different individuals with the same diagnosis. Aberrations may also be found in organs other than the orofacial complex.

TCS is a rare autosomal-dominant condition, mainly caused by mutations in the *TCOF1* gene. The suggested minimal criteria for the diagnosis are downward-slanting palpebral fissures and hypoplasia of the zygomatic arches [1]. Other frequent findings include hypoplasia of the mandible, cleft palate, lower eyelid coloboma, microtia, atresia of the ear canal, and hearing loss. No study has reported an association between the locations of the mutations and the expression of the TCS phenotype [1, 2]. It is estimated that 60 % of cases arise from de novo mutations [3]. Identical mutations in the same family may cause variable expressivity, and there are indications of increased severity over generations [1]. Generally, there is need of management strategies lack multidisciplinary follow-up and treatment planning for respiration, hearing, skeletal growth, and orthodontic issues [4]. Recent findings have indicated that adults with TCS may require multidisciplinary health services due to persistent sleep apnea, compromised oral health, and speech deficits [5–9].

Cherubism is a rare autosomal-dominant condition caused by mutations in the *SH3BP2* gene [10]. It is characterized by symmetrical enlargement of the jaws in children, caused by the replacement of bone with fibrous tissue, which results in a rounded face. In the most severe cases, the orbital floor is affected, which results in upward-looking eyes; these features give the appearance of a cherub, as depicted in baroque paintings. Missing teeth, displaced teeth, and premature tooth loss are additional characteristics [11–13]. Diagnosis is typically based on a combination of clinical, radiographic, histological, and molecular findings, combined with a positive family history. The condition, which is normally painless, typically presents around 3 years of age. The facial swelling increases until puberty [14] and then slowly resolves. According to Meng et al. [15], the facial contours are often unremarkable on clinical examination by the age of 30 years. However, some cases do not resolve with age [13]. According to a recent review article [16], surgical treatment (curettage, contouring, or resection) is not indicated in mild cases, but may be necessary after puberty in moderate cases, during puberty in severe cases, or earlier when severe functional problems are present. Other treatment options, like medication with calcitonin and interferon, may be useful in the future.

The term oligodontia describes the congenital absence of six or more permanent teeth, third molars excluded [17]. Oligodontia is associated with a delay in the development of other teeth and a relative lack of alveolar growth. Most individuals with oligodontia do not have other medical symptoms or findings [18]. Oligodontia is common in many forms of ED. The EDs comprise a large, clinically and etiologically heterogeneous group of genetic disorders; at least 186 distinct pathological conditions have been recognized [19]. No universally accepted classification for EDs has been achieved, but the clinical classification proposed by Freire-Maya [20] is widely used. It is based on the “classical signs” of ED, as follows: (group A) abnormalities in hair, teeth, nail, and sweat glands (two findings or more) and (group B) defects in one of the mentioned structures, plus at least one other ectodermal defect. Individuals with EDs may exhibit deviations in orofacial functions such as chewing, swallowing, and speech [21]. The orofacial symptoms of EDs overlap with those of oligodontia. Both conditions may alter appearance, and salivary secretion is often reduced [22, 23]. The etiologies of the conditions are also overlapping, because the gene mutations that cause full blown EDs may also cause isolated oligodontia [23]. Oral treatment may be challenging, and evaluation by a multidisciplinary team is frequently necessary for optimal treatment planning for individual patients. Achieving an ideal occlusion is not always possible [24].

Former studies on psychological distress and quality of life (QoL) associated with orofacial disorders have mostly focused on children and adolescents. Facial appearance after reconstructive surgery in TCS was shown to have a direct positive influence on psychosocial factors [25], as well as the researchers reported that some children with TCS showed elevated anxiety compared to unaffected children [25]. Another study demonstrated that TCS was associated with increased social anxiety and dissatisfaction [26]. In studies among children and adolescents with EDs, those aged 15–19 years showed worse oral health-related QoL in the subscale “functional limitations,” compared to those aged 10–14 years. Moreover, females experienced more emotional problems than males [27]. In children with hypohidrotic forms of ED, QoL was influenced most by the reduced sweating [28]. Hypodontia, per se, has been found to impact QoL in children, because it results in functional limitations, which reduce social and emotional well-being [29]. In adults, QoL was most affected by actual symptoms and findings of EDs, and the emotional and social consequences had less impact [28]. Physical attractiveness is known to contribute to a positive self-concept and social well-being [26, 30–33]. In the worst cases of EDs, the face has an edentulous appearance, which can lead to considerable psychological disturbance [34].

To our knowledge, few researchers have examined psychological distress and QoL in adults with orofacial disfigurements, and no studies have compared adults affected by different conditions. Therefore, this study aimed to examine and describe differences in psychological distress and QoL between groups of individuals affected by TCS, cherubism, and oligodontia/EDs. We hypothesized that living with facial disfigurement would affect QoL, well-being, and mental health. In light of the differences in congenital orofacial disfigurements, we anticipated that the TCS group may experience more psychological distress and worse QoL than the other two groups.

## Materials and methods

### Participants

#### TCS

Between December 2008 and May 2009, we invited all individuals with TCS that had registered at the National Resource Centre for Oral Health in Rare conditions (TAKO-center), the Centre for Rare Disorders (SSD), and the Craniofacial team at Oslo University Hospital, Rikshospitalet (*n* = 36) to participate in a study. The study included an extensive health examination and the completion of questionnaires on psychological distress and QoL [6, 7]. Twenty-three individuals (64 %) with TCS accepted the invitation. Of these, 10 participants were under 18 years of age, and therefore, they were not included in this part of the study. Two of the remaining 13 responders were excluded, due to an unconfirmed TCS diagnosis [7]. Thus, this study included a total of 11 adults (65 %) aged >18 years. Among these, four had mild and seven had severe TCS phenotypes, according to the severity scoring system developed by Teber et al. [1, 7]. Eight participants had undergone reconstructive surgery in the eye, ear, and/or zygomatic regions [6]; six participants had received orthodontic treatment. Seven patients presented with moderate to profound hearing impairments [8]. Polysomnography demonstrated sleep apnea in all eleven participants [7]. Facial asymmetry was identified by assessing orofacial function with the Nordic Orofacial Test Screen (NOT-S) [6]. Minor speech aberrations were common [8].

#### Cherubism

We previously identified 28 individuals in Norway with cherubism. They were partly known at the TAKO-centre (15) or through family members (6) and partly recruited by contacting all oral and maxillofacial surgeons and orthodontists in Norway (7). Of these 28 patients, 26 underwent genetic screening, and 24 tested positive for mutations in *SH3BP2*. Among these individuals with genetically verified cherubism, 22 participated in a study where they had provided a patient history and attended oral, clinical, and radiologic examinations. They also completed forms for assessing psychological distress and QoL. All participants underwent a full day of examinations, interviews, and clinical genetics consultations in Oslo between September 2009 and December 2011 [13]. Of these 22 individuals, 15 (68 %) were aged >18 years and were included in the present study. Many of these patients had received extensive surgical and dental treatments; 13 patients had undergone one or more surgical procedures for tooth removal, exposure, or transplantation; curettage of intra-bony soft tissue; or contouring; 11 patients had tooth replacements (removable or fixed dentures or dental implants).

#### Oligodontia/ED

All individuals aged 16 years or older that had registered with ED and/or oligodontia at the TAKO-centre were invited to participate in a multidisciplinary study that included questionnaires on QoL and psychological distress. Out of 54 responders (66 %), 49 (91 %) were aged ≥18 years and were included in this study. All individuals lacked a minimum of six teeth, excluding third molars. Other affected ectodermal tissues included skin (*n* = 27; 55.1 %), hair (*n* = 20; 40.8 %), nails (*n* = 19; 38.8 %), and sweat glands (*n* = 10; 20.4 %). In the oligodontia/ED group, 33 of 49 adults had received tooth replacements.

### Demographics

We recorded demographic characteristics including age, family situation, educational level, and work status.

### Psychological distress

#### Hospital anxiety and depression scale (HADS)

The HADS measures self-reported levels of distress [35]. It was originally developed to identify cases of anxiety and depression among patients in non-psychiatric hospitals [36]. It is a 14-item questionnaire with two subscales: anxiety (HADS-A) and depression (HADS-D). Each subscale has seven items rated on a 4-point Likert-style scale from 0 (not present) to 3 (maximally present). The HADS-A and HADS-D score sums ranged from 0 to 21. Based on receiver operating characteristics, a sum ≥8 on each subscale was used as a cutoff score to identify cases of possible anxiety and depression disorders, which may require further clinical examination [35, 37]. A previous study demonstrated that the psychometric properties of the Norwegian version of the HADS were excellent [38].

### Quality of life

#### Overall quality of life

Cantril’s Self-Anchoring Ladder (CL) is a self-administered questionnaire that assesses overall QoL with one question: “How is your life?”. It asks respondents to rate their present experiences with life on a scale anchored by their own identified values. The response alternatives are between 0 and 10, with 0 = worst possible QoL and 10 = best possible QoL [39].

The psychosocial well-being questionnaire, or Kaasa’s test, assesses psychosocial experience of well-being. It consists of five positive and five negative statements, rated from 1 (highest QoL) to 5 (lowest QoL) [40]. In addition, the test includes two single items of overall QoL, happiness and satisfaction, with the ranges of 1 (highest) to 7 (lowest).

#### Health-related quality of life

The Short Form 36 (SF-36) [41] is a multipurpose, generic, self-administered, health-related QoL questionnaire. It includes 36 items rated on eight scales (aggregating 2–10 items each) and the two summary measures Physical Component Scale (PCS) and Mental Component Scale (MCS). The four PCS scales are physical functioning (PF), role limitation owing to physical health problems (RP), bodily pain (BP) which addresses the degree of pain interfering with participation in various activities of everyday life, and general health (GH).The vitality (VT), social functioning (SF), role limitations due to emotional problems (RE), and mental health (MH) comprise the MCS scale.

According to standard SF-36 scoring, all information was transformed to a scale of 0 (worst) to 100 (best). Based on T-transformations, both PCS and MCS have a mean of 50 and a standard deviation of 10 in the US general population. An individual with a score <40 on both PCS and MCS is defined as a case of poor QoL that requires treatment or help in improving the QoL [41]. The SF-36 scale has been extensively validated [42, 43].

### Statistics

Significant differences between groups in demographic variables, psychological distress, and QoL were analyzed, depending on the distribution of variables, with parametric tests (*t* test, one-way ANOVA, pairwise comparisons with Bonferonni test) or with nonparametric tests (Kruskal–Wallis, Mann–Whitney *U*). The TCS and cherubism groups showed normal distribution curves on all dependent variables. The oligodontia/ED group showed minor deviations on a few of the dependent variables. However, these deviations had low impact, due to the sufficiently large size of the oligodontia/ED group; therefore, all variables were analyzed with parametric tests. The nonparametric tests were used for groups with small sample sizes (TCS and cherubism groups) and when any deviations were found in the normal distribution curves. Thus, the Kruskal–Wallis one-way analysis of variance (ANOVA) was used to analyze between-group differences. When significant differences were found, pairwise comparisons were performed with the Mann–Whitney *U* test. However, the latter test did not affect the results; therefore, the results are presented based on the parametric tests only. Pearson’s chi-squared statistic was used to test for significant differences between categorical variables. To estimate the reliability of test scores from the scale measures for the sample of responders, we evaluated the internal consistency with Cronbach’s coefficient *α.* We considered Cronbach’s coefficient *α* values >0.60 acceptable, >0.70 good, and >0.90 excellent. The analyses were performed with PASW Statistics 20.0 for Windows (SPSS Inc, Chicago, IL). The level of statistical significance was set at *p* *≤* 0.05.

### Ethics

This study recruitment procedures and protocol on individuals with cherubism, TCS, and oligodontia/ED were approved by the Norwegian Regional Committee of Ethics in Medical Research as well as the Norwegian Data Inspectorate at Oslo University Hospital. All participants gave written informed consent prior to inclusion in the study.

## Results

Demographics are shown in Table 1. Individuals with oligodontia/ED were younger than those with TCS and cherubism, but no other significant differences were established.

**Table 1:** Demographics

	TCS *N* = 11	Cherubism *N* = 15	Oligodontia/ED *N* = 49
Age, years (mean/SD)	46.8 (12.9)^##^	50.3 (16. 8)*	32.2 (12.9)*
Civil status, *N* (%)			
Paired relation	7 (58)	9 (60)	20 (41)
Non-paired relation	4 (42)	6 (40)	29 (59)
Educational level, *N* (%)			
≤12 years	5 (42)	8 (53)	27 (55)
≥13 years	6 (58)	7 (47)	22 (45)
Employment, *N* (%)			
Yes	8 (67)	12 (75)	26 (53)
No	3 (33)	3 (25)	23 (47)

*TCS* Treacher Collins syndrome, *ED* ectodermal dysplasia

* *p* < 0.001, significant differences between cherubism and oligodontia/ED

^##^ *p* < 0.01, significant differences between TCS and oligodontia/ED

### Psychological distress (HADS)

One-way ANOVAs revealed statistically significant differences among the three different groups in anxiety and depression; the Bonferonni analysis suggested that the oligodontia/ED group had a significantly higher level of anxiety than both the other groups. Among the three groups, the TCS group showed the highest level of depression (Table 2). Cronbach’s *α* for oligodontia/ED, TCS, and cherubism were 0.70, 0.73, and 0.71, respectively, for the HADS-A; and 0.73, 0.70, and 0.65, respectively, for the HADS-D. These estimations indicated good internal consistency.

**Table 2:** Differences in anxiety and depression across groups

	TCS		Cherubism		Oligodontia/ED		*p* ^a^
	Mean	SD	Mean	SD	Mean	SD	
HADS-A	3.18	2.71	2.46	2.11	6.96^§§,###^	3.73	<0.001
HADS-A cases, *N* (%)	1 (9)		0		25 (51)		
HADS-D	4.53^#^	3.44	0.85	1.34	3.51^#^	3.47	<0.001
HADS-D cases, *N* (%)	1 (9)		0		6 (12)		

*HADS-A* hospital anxiety and depression anxiety score-anxiety, *HADS-D* hospital anxiety and depression score-depression, *TCS* Treacher Collins syndrome, *ED* ectodermal dysplasia

^a^ *p* was based on ANOVA

^#^ *p* < 0.05; ^###^ *p* < 0.001 compared to cherubism

^§§^ *p* < 0.01 compared to TCS

### Quality of life

#### Overall QoL and psychosocial well-being

Table 3 reveals that the cherubism group had significantly better overall QoL, satisfaction, and happiness than both the TCS and oligodontia/ED groups. The poorest well-being was found among adults with TCS.

**Table 3:** Differences in quality of life across groups

	TCS		Cherubism		Oligodontia/ED		*p* ^a^
	Mean	SD	Mean	SD	Mean	SD	
Overall quality of life							
Cantril’s self-anchoring ladder (CL)	6.36	2.58	9.00^§§,^**	0.86	7.37	1.94	0.002
Psychosocial well-being							
Kaasa’s test							
Well-being	3.76***^,##^	0.77	2.82	0.14	2.29	0.72	<0.001
Happiness	2.82	0.60	1.77*	0.60	2.66	1.27	0.027
Satisfaction	2.36	0.67	1.32*	1.62	2.55	1.32	0.360
Health-related quality of life							
SF-36							
Physical functioning	77.72*^,#^	19.55	92.33	14.86	91.63	14.12	0.021
Role physical	90.63	18.60	91.07	21.05	85.20	24.16	Ns
Bodily pain	30.65*^,#^	9.24	86.07	18.71	75.87	27.58	0.030
General health	69.36	17.57	78.88	19.30	73.97	24.23	Ns
Vitality	54.54	15.57	63.78	12.76	58.30	24.79	Ns
Social functioning	81.81	26.43	96.97	12.89	82.81	27.72	Ns
Role emotional	93.80	26.43	96.66	12.90	65.78^§§§,###^	18.34	<0.001
Mental health	81.09	14.43	82.20	14.67	74.45	20.16	Ns
Physical component scale (PCS)	42.97**^,##^	10.30	52.1	8.56	52.93	7.92	0.003
PCS case, *N* (%)	4 (40)		2 (13)		5 (10)		
Mental component scale (MCS)	54.20	9.55	54.29	5.42	46.24^§§,##^	8.26	0.001
MCS case, *N* (%)	1 (9)		0		11 (23)		

*TCS* Treacher Collins syndrome, *ED* ectodermal dysplasia

^a^ *P* was based on ANOVA

Ns = nonsignificant, based on ANOVA

* *p* < 0.05; ** *p* < 0.01; and *** *p* < 0.001, compared to oligodontia/ED

^#^ *p* < 0.05; ^##^ *p* < 0.01; ^###^ *p* < 0.001, compared to cherubism

^§§^ *p* < 0.01; ^§§§^ *p* < 0.001, compared to TCS

Kaasa’s test showed good internal consistency, with Cronbach’s *α* values of 0.84, 0.74, and 0.87 for TCS, cherubism, and oligodontia/ED, respectively.

#### Health-related QoL

Significant differences in health-related QoL were found among the groups in physical functioning, bodily pain, emotional-role limitations, and in the component scores, PCS and MCS. The TCS group showed the poorest physical functioning, bodily pain, and PCS, and the oligodontia/ED group showed the poorest emotional-role limitations and MCS (Table 3).

The SF-36 results showed good internal consistency, with Cronbach’s *α* values between 0.72 (mental health) and 0.89 (emotional-role limitations) in the TCS group; 0.61 (bodily pain) and 0.87 (physical-role limitations) in the cherubism group; and 0.74 (bodily pain) and 0.93 (physical-role limitations) in the oligodontia/ED group. These values were considered acceptable in all three groups.

## Discussion

To our knowledge, this was the first study to compare psychological distress and QoL among adults with three different genetic conditions that affected the orofacial complex. Our main findings were that psychological distress and QoL differed among the various orofacial conditions. Individuals with TCS reported the most severe depression, the least well-being, and worst physical QoL. On the other hand, the oligodontia/ED group showed the highest level of anxiety and the poorest mental QoL. The cherubism group showed the best overall QoL and experienced the most satisfaction and happiness.

Cherubism develops in childhood and improves over time, in the sense that the condition changes to a near normal appearance in most adults. In contrast, TCS and oligodontia/ED are permanent states of congenital facial disfigurement. The disfigurement in TCS is frequently worse than that observed in oligodontia/ED. In both diagnoses, advanced interdisciplinary surgical and dental treatment may improve oral functions over time. For example, the replacement of missing teeth and alveolar bone are often performed in oligodontia/ED, and multiple corrections of micrognathia and malocclusion are common in TCS. However, these treatments are long-term processes and may cause psychological burden. EDs may also be associated with other dysfunctions, such as the lack of a capacity to sweat, low salivary secretion, and dry eyes. TCS is also accompanied by physical symptoms, like reduced salivary secretion, which can affect oral health and eating [5, 6]. In addition, TCS is characterized by congenital hearing loss in moderate- to-severe degree [8].

The TCS group showed significantly higher levels of depression compared to the two other groups. Depression is often a response to loss. For a person with orofacial disfigurement, loss may be related to the absence of a “normal” appearance. According to Hatfield and Sprecher [44], physical appearance is important in the development of interpersonal relationships, and physical attractiveness is known to contribute to a positive self-concept and social well-being [31]. Based on discussions and clinical experience, individuals with TCS, from time to time, experienced glaring eyes and sometimes rejection, due to their facial disfigurement. These factors may partly explain why the TCS group experienced worse overall QoL and well-being than the other groups. QoL factors, like satisfaction with life, happiness, and a feeling of well-being, are subjective terms that refer to the individual achievement of one’s goals [45]. A gap between one’s expectations and the reality of life may cause depression, and thereby, reduce the QoL. The higher level of depression in the TCS group may indicate that living with TCS includes more obstacles to participating in everyday life than living with the other two conditions.

It was previously reported that all adults of the current TCS study group had obstructive sleep apnea (OSA) [7]. More than 70 % had moderate-to-severe OSA, and the severity of OSA was significantly associated with reduced physical health-related QoL [9]. This association may explain the report that physical health-related QoL was worse in the TCS group than in the oligodontia/ED and cherubism groups.

The oligodontia/ED group showed the highest level of anxiety. Anxiety is related to worry and uncertainty about the future. The fact that more than 50 % of individuals with oligodontia/ED had anxiety scores above the level that requires treatment may provide insight into this condition. The differences among the groups may be related to differences in stressors; thus, adults in the oligodontia/ED group may experience a different type of stress than adults in the other groups. For example, many individuals with oligodontia/ED have noticeable spaces between the teeth, which may cause significant teasing and bullying in adolescence [46]. At some stage in adulthood, these individuals often undergo extensive prosthodontic treatment. However, Norway lacks a coordinated approach for offering appropriate dental services to individuals with oligodontia/ED. A qualitative study performed in Ireland has shown that delays in completing dental treatment caused considerable frustration in patients with oligodontia/ED [47]. In another study that included children and adolescents with TCS, the main findings showed that improved facial appearance after reconstructive surgery had a positive influence on psychosocial and social factors [27]. This may also apply to adults with TCS; in most cases, the adults in the present study had completed surgical treatments, and hence, they were more likely to experience less anxiety than individuals in the oligodontia/ED group.

In addition to the high level of anxiety, the oligodontia/ED group presented the poorest mental health-related QoL. This finding was consistent with results from Mehta et al. [48], who found that emotional distress was highly prevalent in adults with ED. Hypodontia, per se, was found to impact QoL in children, because it resulted in functional limitations and reduced social and emotional well-being [29]. Furthermore, Anweigi et al. [49] reported that non-syndromal hypodontia had a substantial impact on oral health-related QoL and that functional limitations increased with age. In the present study, the mean age of individuals in the oligodontia/ED group was significantly younger than that of individuals in the other groups; thus, it is possible that we may have underestimated the impact of this condition on psychological distress and QoL.

Few studies are available that investigated psychological distress and QoL in individuals with cherubism. In a recently published study, Prescott et al. [13] found that QoL among individuals with cherubism was not significantly different from QoL in an age and gender-adjusted sample of the general Norwegian population. In the present study, individuals with cherubism reported the least psychological distress and the highest level of QoL among the three groups. Thus, we confirmed the findings from Prescott et al. that individuals with cherubism appear to be well-adjusted on a group level. However, it is important to offer individuals with cherubism personalized healthcare services.

One strength of this study was that we used established, validated, self-rating measures. The three conditions studied are rare, and therefore, another strength was that we invited all registered adult individuals with TCS and cherubism in Norway to participate in the study. It was relatively easy to recruit these individuals through national registry services. Because oligodontia/ED comprises a larger group than TCS and cherubism, treatment is offered by many different institutions. Hence, there was no single registry that contained a complete listing of individuals with oligodontia/ED in Norway. All patients registered at the TAKO-centre with oligodontia/ED were invited to participate, and patients from all parts of Norway responded. This recruitment approach yielded an adequate sample; moreover, we assume that our findings can be generalized to all Norwegian individuals with these conditions. However, the study had some limitations. It was a cross-sectional study; thus, our results revealed important knowledge about various aspects of psychological distress and QoL related to a particular disease status, but we could not determine whether these parameters might change over time.

### Clinical implications

We found that living with orofacial disfigurement caused psychological distress, and it reduced the QoL to varying extents among the different groups. Social and healthcare services should focus on alleviating the high level of anxiety and low mental QoL in adults with oligodontia/ED; moreover, management strategies should include genetic counseling. In addition, healthcare services should focus on alleviating depression and low physical QoL in individuals with TCS.

## Conclusions

This study showed that psychological distress and QoL differed among individuals with TCS, cherubism, and oligodontia/ED. The oligodontia/ED group was characterized by a high level of anxiety and reduced mental QoL. The TCS group experienced a high level of depression and reduced physical QoL. Finally, the cherubism group experienced less psychological distress and better QoL than the other two groups included in this study.
